# Significance of Microvascular Function in Visual—Functional Mismatch Between Invasive Coronary Angiography and Fractional Flow Reserve

**DOI:** 10.1161/JAHA.117.005916

**Published:** 2017-05-31

**Authors:** Taishi Yonetsu, Tadashi Murai, Yoshihisa Kanaji, Tetsumin Lee, Junji Matsuda, Eisuke Usui, Masahiro Hoshino, Makoto Araki, Takayuki Niida, Masahiro Hada, Sadamitsu Ichijo, Rikuta Hamaya, Yoshinori Kanno, Tsunekazu Kakuta

**Affiliations:** ^1^ Department of Cardiovascular Medicine Tsuchiura Kyodo General Hospital Tsuchiura Ibaraki Japan

**Keywords:** angiography, coronary artery disease, fractional flow reserve, microvascular dysfunction, percutaneous coronary intervention, Coronary Circulation, Angiography, Catheter-Based Coronary and Valvular Interventions

## Abstract

**Background:**

Despite a moderate correlation between angiographical stenosis and physiological significance, the mechanism of discordance has not been fully elucidated, particularly regarding the significance of microvascular function. This study sought to clarify whether microvascular function affects visual‐functional mismatch between quantitative coronary angiography (QCA) and fractional flow reserve (FFR).

**Methods and Results:**

We assessed QCA, FFR, coronary flow reserve, and the index of microcirculatory resistance in 849 non‐left‐main coronary lesions with visually estimated intermediate stenoses from 532 patients. Clinical and lesion‐specific characteristics and physiological parameters associated with mismatch and reverse mismatch were studied. Coronary flow reserve and index of microcirculatory resistance showed a weak, but significant, correlation with FFR (R=0.306, *P*<0.001 and R=0.158, *P*<0.001, respectively). Four hundred twenty‐two lesions were visually nonsignificant (diameter stenosis assessed by QCA [QCA‐DS] ≤50%) and 427 lesions were visually significant (QCA‐DS >50%). Among visually nonsignificant lesions, FFR ≤0.80 (reverse mismatch) was observed in 129 lesions (30.6%). Among visually significant lesions, FFR >0.80 (mismatch) were observed in 179 lesions (41.9%). The significant predictors of reverse mismatch were male sex, nonculprit lesions of acute coronary syndrome, left anterior descending artery location, smaller QCA reference diameter, greater QCA‐DS, lower coronary flow reserve, and lower index of microcirculatory resistance. Mismatch was associated with right coronary artery location, greater QCA reference diameter, smaller QCA‐DS, lesion length, higher coronary flow reserve, and higher index of microcirculatory resistance.

**Conclusions:**

There was a high prevalence of visual‐functional mismatches between QCA and FFR. The discrepancy was related to clinical characteristics, lesion‐specific factors, and microvascular resistance that was undistinguishable by coronary angiography, thus suggesting the importance of physiological lesion assessment.

**Clinical Perspective**

**What is New?**

- High coronary flow reserve and high index of microcirculatory resistance were associated with visual‐functional mismatch in which angiographic diameter stenosis exceeds 50% and FFR was not indicative of ischemia (FRR >0.80).
- Low CFR and low IMR were associated with visual‐functional reverse mismatch in which angiographic diameter stenosis was less than 50% and FFR suggested the presence of ischemia (FFR≤0.80).

**What are the Clinical Implications?**

- Visual‐functional mismatch and reverse mismatch are not rare.
- In addition to clinical characteristics and lesion location, microvascular function may play important roles for visual‐functional mismatch and reverse mismatch, which corroborates the importance of coronary flow physiological assessment.

## Introduction

In addition to clinical characteristics and lesion location, microvascular function may play important roles for visual‐functional mismatch and reverse mismatch, which corroborates the importance of coronary flow physiological assessment.

Invasive coronary angiography is a well‐accepted method for identifying the presence of flow‐limiting epicardial coronary artery stenosis and for guiding revascularization. The seminal study by Gould et al reported that hyperemic flow begins to decline in the presence of stenosis with a reduction in diameter larger than 50%.^1^ This cut‐off value has been used for the threshold of inducible ischemia; therefore, it is accepted as the gold standard for guiding revascularization, validating noninvasive testing, and evaluating outcomes after revascularization strategies.^2^, ^3^, ^4^ However, cumulative evidence suggests that angiographically determined anatomical stenosis severity often underestimates or overestimates the functional significance of lesions.^5^, ^6^, ^7^ Fractional flow reserve (FFR) is currently the standard for decision‐making regarding revascularization in the catheter laboratory and has become part of the clinical guidelines for the assessment of the physiological significance of epicardial coronary stenosis based on sound concepts and randomized clinical trials.^8^, ^9^, ^10^, ^11^ However, FFR evaluation is still underutilized; instead, coronary angiography is widely used as a gatekeeper for decision‐making of revascularization even in large clinical trials.^2^, ^12^ Subanalysis of the FAME (Fractional Flow Reserve Versus Angiography for Multivessel Evaluation) trial has shown that angiography is inaccurate for assessing the functional significance of coronary stenosis when compared with FFR guidance, especially for intermediate stenosis.^6^ Although such “visual‐functional mismatch” or “reverse visual‐functional mismatch” are not uncommon, the mechanism of these phenomena has not been fully elucidated. Recent studies further specifically suggest the relevance of microvascular function to FFR.^13^, ^14^, ^15^ Visual‐functional mismatch has important implications in overcoming limitations related to angiography‐guided decision‐making to reduce unnecessary revascularization and avoiding missed appropriate revascularization. Therefore, we sought to identify lesion‐specific, patient‐related factors associated with visual‐functional mismatch using a large cohort of the institutional database that includes FFR, coronary flow reserve (CFR), and the index of microcirculatory resistance (IMR) information with a particular focus on microvascular function.

## Methods

### Study Population

**Figure 1:**
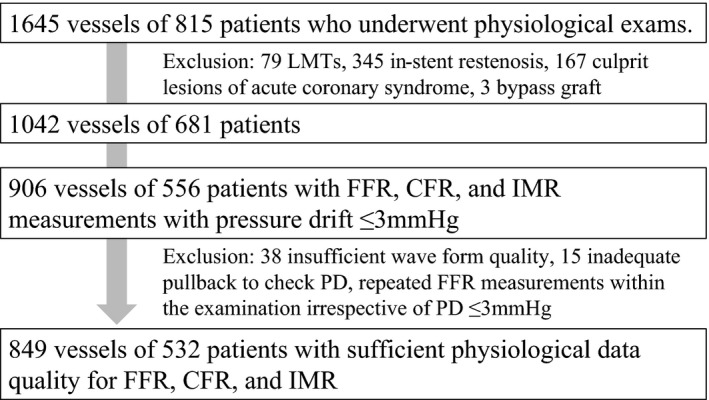
Patient population. CFR indicates coronary flow reserve; FFR, fractional flow reserve; IMR, index of microcirculatory resistance; LMT, left main trunk disease; PD, pressure drift.

A retrospective analysis of pooled data was performed in the institutional FFR registry listing pressure and ECG tracings between December 2010 and May 2016 that included consecutive 1645 FFR measurements of 815 patients referred for diagnostic or therapeutic catheterization. Institutional indication criteria for coronary physiological assessment including FFR, CFR, and IMR were intermediate coronary stenosis showing 30% to 80% diameter stenosis by visual estimation in patients with stable angina pectoris; in those with suspected asymptomatic ischemia and/or microvascular dysfunction; or in nonculprit lesions of acute coronary syndrome (ACS) more than 2 weeks after onset. We selected 1042 eligible lesions from 623 patients who met the following criteria (Figure 1): age older than 20 years; detection of an identifiable de novo lesion located at the proximal to middle portion of a native coronary artery; and patients with stable coronary disease or those with ACS in whom the culprit lesions were treated >2 weeks before the examination. Patients with a history of coronary artery bypass surgery, culprit lesions of ACS, left main disease, and in‐stent restenosis were excluded from the analysis. Although left main disease has been reported to be an important factor in reverse visual‐functional mismatch,^16^ the present study excluded those lesions because the relationship between left main disease and microvascular function is not able to be assessed by the IMR. In those 1042 eligible lesions, we further identified lesions with sufficient physiological data for the determination of FFR, CFR, and IMR. Insufficient acquisition of physiological data, such as pressure drift (PD) >3 mm Hg, insufficient waveform during the examination, and repeated FFR measurements irrespective of PD within 3 mm Hg, were excluded. The study was approved by the local ethics committee and conformed to the Declaration of Helsinki statement on research involving human subjects. Informed consent was provided by all participants after a complete explanation of the protocol and potential risks.

### Cardiac Catheterization

Each patient initially underwent standard selective coronary angiography through the radial artery using a 6‐Fr system. Quantitative analyses of angiograms were performed using offline analysis software (QAngio XA; Medis Medical Imaging Systems, Leiden, The Netherlands) to measure minimum lumen diameter, (QCA) reference lumen diameter (QCA‐RD), QCA percent diameter stenosis (QCA‐DS), and lesion length of the target lesion. All patients received a bolus injection of heparin (5000 IU) before the procedure and an additional bolus injection of 2000 IU every hour if the procedure required more than 1 hour. An intracoronary bolus injection of nitroglycerin (0.2 mg) was administered at the beginning of the procedure and repeated every 30 minutes. Intravenous infusion of ATP (150 μg/kg/min) was used to induce hyperemia. According to the manufacturer's specifications, which reported an acceptable pressure error range of 3%, we analyzed FFR measurements with PD ≤3 mm Hg in the present study. Our institutional standard protocol recommends repeated FFR measurements when PD ≥4 mm Hg; however, the final decision to repeat the measurements is at the discretion of the operator. Lesions with PD >3 mm Hg were excluded from the analysis, and FFR was calculated as a corrected value of distal coronary pressure (Pd)/proximal coronary pressure (Pa) after correction of Pd by PD.

### Intracoronary Physiological Indices

FFR, CFR, and IMR were measured with a RadiAnalyzer Xpress instrument and a PressureWire Certus (pressure‐temperature sensor wire; St. Jude Medical, Uppsala, MN).^5^, ^17^, ^18^ The pressure wire was introduced, zeroed, and equalized to the catheter tip pressure before crossing the lesion. Afterward, the pressure sensor was positioned 8 to 10 cm distal to the ostium of the studied artery across the lesion. Baseline pressures were recorded for at least 20 seconds after the guiding catheter was flushed with saline. Thereafter, FFR measurements were performed at stable hyperemia. PD was determined when the pressure sensor was pulled back and reached the tip of the guiding catheter. All pressure and ECG tracings in the catheterization laboratory's monitoring system (RMC‐4000 Cardio Master with EP amplifier system JB400G; Nihon Koden, Tokyo, Japan) were submitted to the in‐hospital core cardiac physiology and morphological analysis laboratory. Insufficient waveform quality, including absence of pressure signal, catheter‐damped waveform, and inappropriate Pd waveform, were excluded. Analyzed FFR data were compared with the original readout values determined by the catheterization laboratory; a consensus reading was agreed on by 2 expert physicians if there were discordant values. In the present study, IMR was calculated as the product of the mean distal coronary pressure during stable hyperemia and mean hyperemic transit time (Tmn) and corrected by using the following formula proposed by Yong et al^19^: IMR=Pa×Tmn×([1.35×Pd/Pa]–0.32). In the absence of a validated cutoff to identify abnormally increased hyperemic microvascular resistance and the reported variability of IMR in patients with or without coronary heart disease, IMR values ≥75th percentile (28.0) of the present cohort were arbitrarily assumed as high IMR.^20^, ^21^ CFR was also measured simultaneously with FFR using the thermodilution method, as described elsewhere.^18^ In this study, low CFR was defined as a value <2.0, consistent with previous studies.^22^

### Definition of Visual‐Functional Reverse Mismatch and Mismatch

The clinical cut‐off point of functionally significant ischemia was defined as FFR of 0.80. Visually significant stenosis was defined as QCA‐DS >50%. Lesions were categorized into 4 groups according to the FFR and QCA‐DS thresholds: concordantly nonsignificant group showing QCA‐DS ≤50% and FFR >0.80; visual‐functional reverse mismatch group; visual‐functional mismatch group; or concordantly significant group showing QCA‐DS >50% and FFR ≤0.80. Visual‐functional reverse mismatch was defined as QCA‐DS ≤50% and FFR ≤0.8. Visual‐functional mismatch was defined as QCA‐DS >50% and FFR >0.80. Determinants of reverse mismatch in visually nonsignificant stenosis and those of mismatch in visually significant stenosis were investigated in the present study.

### Statistical Analysis

Statistical analysis was performed by using SPSS (version 23.0; SPSS Inc, Chicago, IL) and R software (version 3.2.3; R Foundation for Statistical Computing, Vienna, Austria). Patient demographics are presented as n (%), when appropriate. Categorical data are expressed as absolute frequencies and percentages and were compared using χ^2^ or Fisher's exact tests, as appropriate. Correlations between FFR and QCA data, CFR, and IMR were assessed by Pearson correlation analysis. Data were analyzed on a per‐patient and per‐lesion basis for the corresponding calculations. For per‐patient data, continuous variables are expressed as mean±SD for normally distributed variables or as median (25th–75th percentile) for non‐normally distributed variables and compared using Student *t* tests and Mann–Whitney *U* tests, respectively. For per‐lesion data, a logistic generalized estimated equation model with robust SEs that accounted for the clustering between lesions in the same subject was created. A multivariable logistic regression analysis was performed to identify the determinants for QCA‐FFR visual functional reverse mismatch and mismatch; results were presented as odds ratios (ORs) and 95% CI. The associated variables in univariate analysis (*P*≤0.20) and physiological parameters, including CFR and IMR, were entered into the final multivariable model. *P*<0.05 was considered statistically significant.

## Results

### Patient Characteristics and Angiographic, Procedural, and Hemodynamic Results

**Figure 2:**
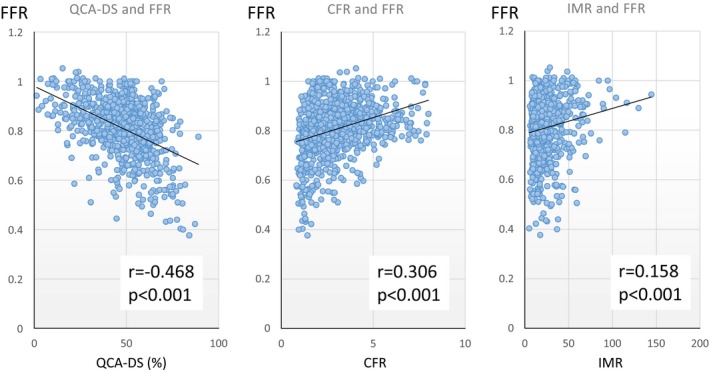
Correlation between FFR and angiographical and physiological indices. CFR indicates coronary flow reserve; FFR, fractional flow reserve; IMR, index of microcirculatory resistance; QCA‐DS, percent diameter stenosis determined by quantitative coronary angiography. QCA‐DS showed moderate correlations with FFR and CFR (left panel). CFR and IMR showed weak, but significant, correlations with FFR (middle and right panels).

Of the 1042 eligible lesions with intermediate stenosis, 906 lesions from 556 patients in whom FFR, CFR, and IMR determinations were performed with a pullback PD ≤3 mm Hg were identified (Figure 1). Thereafter, 57 lesions were excluded from the final analysis because of insufficient waveform quality (n=38), inadequate pullback maneuver to check PD (n=15), or the performance of repeated FFR measurements irrespective of PD within 3 mm Hg (n=4), leaving 849 FFR measurements from 532 patients in the final analysis. Patient and lesion characteristics of the final cohort are summarized in Table 1. Average age was 66.9 years, 82.7% were male, 37.0% had a history of diabetes mellitus, and 71.1% had a history of hypertension. In QCA analysis, mean QCA‐DS was 48.3±14.9% and 427 lesions (50.3%) had QCA‐DS >50%. FFR ≤0.80 was observed in 377 (44.4%) lesions. Reverse mismatch was observed in 129 lesions, which accounted for 15.2% of the total cohort and 30.6% of visually nonsignificant stenosis, whereas mismatch was observed in 179 lesions, accounting for 21.1% of the total cohort and 41.9% of visually significant stenosis (Table 2). In the present cohort, median IMR was 18.0 (12.2–28.1). Lesion characteristics and physiological data of the groups stratified by QCA‐DS and FFR are summarized in Table 2. CFR and IMR were significantly lower in the reverse mismatch group than in the concordantly nonsignificant group, whereas the mismatch group showed higher CFR and IMR as compared with concordantly significant lesions. Correlations of FFR and other parameters, including QCA‐DS, CFR, and IMR, are depicted in Figure 2. QCA‐DS showed a moderate correlation with FFR (correlation coefficient, r=−0.468; *P*<0.001). CFR and IMR showed weak, but significant, correlations with FFR (r=0.306, *P*<0.001 and r=0.158, *P*<0.001, respectively).

**Table 1:** Patient and Lesion Characteristics of Overall Cohort

Patient Characteristics	
n	532
Age	66.9±9.7
Male sex	440 (82.7)
Height, m	1.62±8.9
Weight, kg	64.4±12.3
BMI, kg/m^2^	24.4±3.5
Hypertension	378 (71.1)
Diabetes mellitus	197 (37.0)
Dyslipidemia	309 (58.1)
Current smoker	137 (25.8)
Atrial fibrillation	44 (8.3)
Chronic kidney disease	47 (8.8)
Previous MI	140 (26.3)
Previous PCI	379 (71.2)
Acute coronary syndrome	99 (18.6)
Laboratory data	
CRP, mg/dL	0.42±0.98
BUN, mg/dL	18.0±8.1
Creatinine, mg/dL	0.82 (0.70–0.98)
eGFR, mL/min/1.73 m^2^	68.7±22.2
HbA1c, %	6.3±1.1
LDL‐cholesterol, mg/dL	101±31
HDL‐cholesterol, mg/dL	47±13
Triglyceride	148±106
Medication	
Antiplatelet therapy	385 (72.4)
Calcium blocker	234 (44.0)
Statin	308 (57.9)
Lesion characteristics	
n	849
Lesion location	
RCA	175 (20.6)
LAD	510 (60.1)
Cx	164 (19.3)
Nonculprit lesion of ACS	134 (15.8)
Previous MI‐related artery	84 (9.9)
Quantitative coronary angiography	
Minimal lumen diameter	1.46±0.63
Reference diameter	2.85±0.64
Diameter stenosis	48.3±14.9
Lesion length	12.4±7.6
Physiological data	
Baseline HR, bpm	68 (61–75)
Baseline Pa, mm Hg	93 (84–103)
Baseline Pd, mm Hg	86 (76–96)
Baseline Tmn, s	0.87 (0.59–1.23)
Hyperemic Pa, mm Hg	83 (75–92)
Hyperemic Pd, mm Hg	67 (57–77)
Hyperemic Tmn, s	0.29 (0.20–0.44)
FFR	0.82 (0.75–0.89)
CFR	2.81 (1.89–4.00)
IMR	18.0 (12.2–28.1)

ACS indicates acute coronary syndrome; BMI, body mass index; BUN, blood urea nitrogen; CFR, coronary flow reserve; CRP, C‐reactive protein; Cx, left circumflex artery; eGFR, estimated glomerular filtration rate; FFR, fractional flow reserve; HbA1c, glycated hemoglobin; HDL, high‐density lipoprotein; HR, heart rate; IMR, the index of microcirculatory resistance; LAD, left anterior descending artery; LDL, low‐density lipoprotein; MI, myocardial infarction; Pa, arterial pressure; PCI, percutaneous coronary intervention; Pd, distal pressure; RCA, right coronary artery; Tmn, mean transit time.

**Table 2:** Lesion Characteristics Stratified by Diameter Stenosis and FFR (849 lesions)

	Visually Nonsignificant Lesions QCA‐DS ≤50%			Visually Significant Lesions QCA‐DS >50%		
	FFR>0.80 Concordantly Non‐Significant	FFR≤0.80 “Reverese Mismatch”	*P* Value	FFR>0.80 “Mismatch”	FFR≤0.80 Concordantly Significant	*P* Value
n	293	129		179	248	
Lesion location						
RCA	66 (22.5)	13 (10.1)	<0.001	59 (33.0)	37 (14.9)	<0.001
LAD	165 (56.3)	113 (87.6)		70 (39.1)	162 (65.3)	
Cx	62 (21.2)	3 (2.3)		50 (27.9)	49 (19.8)	
ACS nonculprit	32 (10.9)	30 (23.3)	0.002	33 (18.4)	39 (15.7)	0.513
Infarction‐related vessel	35 (12.7)	14 (11.5)	0.869	15 (8.7)	20 (8.3)	1.000
FFR	0.89 (0.85–0.93)	0.76 (0.70–0.79)	<0.001	0.86 (0.83–0.90)	0.73 (0.64–0.77)	<0.001
CFR	3.07 (2.20–4.18)	2.79 (1.83–4.00)	0.032	3.09 (2.22–4.30)	2.16 (1.43–3.03)	<0.001
IMR	19.3 (12.8–30.5)	15.0 (10.9–21.5)	<0.001	19.2 (12.2–28.3)	17.5 (11.9–28.1)	0.277

ACS indicates acute coronary syndrome; CFR, coronary flow reserve; Cx, left circumflex artery; FFR, fractional flow reserve; IMR, the index of microcirculatory resistance; LAD, left anterior descending artery; RCA, right coronary artery.

### Determinants of FFR

In the overall population, a multivariable linear regression analysis showed that the significant factors affecting FFR as continuous variables were age (coefficient, ß=0.0016; *P*<0.001), QCA‐RD (coefficient ß=0.0358; *P*<0.001), QCA‐DS (coefficient ß=−0.0031; *P*<0.001), CFR (coefficient ß=−0.0223; *P*<0.001), and IMR (coefficient, ß=0.0013; *P*<0.001; Table 3). In addition, multivariable logistic regression analysis showed that the significant determinants for FFR ≤0.80 were male sex (OR, 2.60; 95% CI, 1.51–4.47; *P*=0.001), left anterior descending coronary artery (OR, 4.70; 95% CI, 3.10–7.12; *P*<0.001), smaller QCA‐RD (OR, 0.36; 95% CI, 0.24–0.56; *P*<0.001), greater QCA‐DS (OR, 1.07; 95% CI, 1.05–1.10; *P*<0.001), longer QCA lesion length (OR, 1.03; 95% CI, 1.00–1.06; *P*<0.001), lower CFR (OR, 0.66; 95% CI, 0.56–0.77; *P*<0.001), and lower IMR (OR, 0.98; 95% CI, 0.96–0.99; *P*<0.001; Table 4).

**Table 3:** Determinants of FFR in Multivariate Linear Regression Analysis (849 Lesions)

	95% CI			
	Coefficient ß	Lower	Upper	*P* Value
Age, y	0.0016	0.0012	0.0020	0.001
QCA‐DS, %	−0.0031	−0.0033	−0.0029	<0.001
QCA‐RD, mm	0.0359	0.0309	0.0409	<0.001
CFR	0.0223	0.0200	0.0246	<0.001
IMR	0.0013	0.0011	0.0015	<0.001

CFR indicates coronary flow reserve; FFR, fractional flow reserve; IMR, the index of microcirculatory resistance; QCA‐DS, diameter stenosis assessed by QCA; QCA‐RD, reference diameter assessed by quantitative coronary angiography (QCA).

**Table 4:** Univariate and Multivariate Logistic Regression Analysis for Determinants of FFR ≤0.80 (849 Lesions)

	OR	95% CI		*P* Value	OR	95% CIl		*P* Value
		Lower	Upper			Lower	Upper	
Male	1.75	1.14	2.69	0.011	2.59	1.55	4.35	<0.001
Diabetes mellitus	1.35	0.96	1.89	0.081	1.20	0.82	1.76	0.353
Current smoking	0.70	0.48	1.01	0.059	0.81	0.50	1.31	0.391
ACS nonculprit lesion	1.47	0.97	2.22	0.068	1.52	0.91	2.54	0.111
eGFR, mL/min	0.99	0.99	1.00	0.147	1.00	0.99	1.01	0.926
LDL‐C, mg/dL	1.00	0.99	1.00	0.079	0.99	0.99	1.00	0.057
LAD	2.86	2.07	3.95	<0.001	4.30	2.88	6.44	<0.001
QCA‐RD, mm	0.40	0.31	0.51	<0.001	0.38	0.28	0.51	<0.001
QCA‐DS, %	1.06	1.05	1.07	<0.001	1.07	1.06	1.09	<0.001
QCA‐LL, mm	1.06	1.03	1.09	<0.001	1.03	1.00	1.06	0.021
CFR	0.69	0.61	0.78	<0.001	0.69	0.60	0.80	<0.001
IMR	0.99	0.98	0.99	0.003	0.98	0.96	0.99	<0.001

ACS indicates acute coronary syndrome; CFR, coronary flow reserve; eGFR, estimated glomerular filtration rate; FFR, fractional flow reserve; IMR, the index of microcirculatory resistance; LAD, left anterior descending artery; LDL, low‐density lipoprotein; QCA‐DS, diameter stenosis assessed by QCA; QCA‐LL, lesion length assessed by QCA; QCA‐RD, reference diameter assessed by quantitative coronary angiography (QCA).

### Determinants of Reverse Mismatch and Mismatch

**Figure 3:**
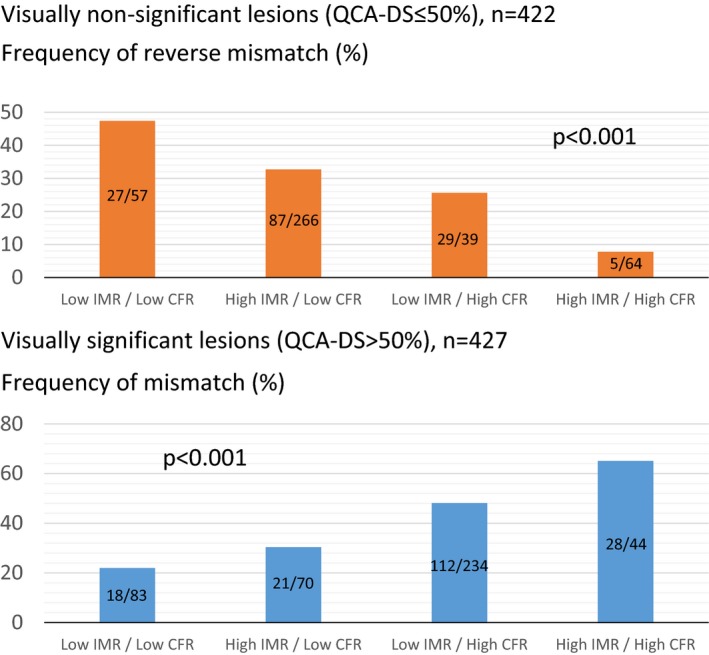
Frequency of visual—functional reverse mismatch and mismatch stratified by IMR and CFR values (lesion‐based analysis). CFR indicates coronary flow reserve; IMR, index of microcirculatory resistance; QCA‐DS, percent diameter stenosis determined by quantitative coronary angiography. Upper panel shows the frequencies of reverse mismatch in visually nonsignificant stenosis in the groups stratified by IMR and CFR thresholds. Reverse mismatch was most frequently observed in the group with low IMR (IMR ≤28.0) and low CFR (CFR <2.0). Lower panel shows the mismatch frequencies in visually significant stenosis in the groups stratified by IMR and CFR thresholds. Mismatch was most frequent in the group with high IMR (IMR >28.0) and high CFR (CFR ≥2.0).

Univariate and multivariable analyses of factors predicting reverse mismatch in visually nonsignificant lesions are shown in Table 5. Multivariable analysis identified male sex, nonculprit lesion of acute coronary syndrome, lower low‐density lipoprotein cholesterol level, left anterior descending coronary artery lesions, smaller QCA‐RD, larger QCA‐DS, lower CFR, and lower IMR as the significant predictors of reverse mismatch in visually nonsignificant lesions. In visually significant lesions, significant predictors of mismatch were right coronary artery, greater QCA‐RD, smaller QCA‐DS, shorter QCA lesion length, higher CFR, and higher IMR (Table 6). Figure 3 shows the frequency of reverse mismatch in visually nonsignificant lesions and that of mismatch in visually significant lesions stratified by IMR (>28.0) and CFR (≤2.0). In visually nonsignificant lesions, frequency of reverse mismatch was significantly different among the 4 groups categorized by IMR and CFR and highest in lesions with low IMR and low CFR values. In contrast, in visually significant lesions, frequency of mismatch was highest in the group with high IMR and high CFR (Figure 3).

**Table 5:** Predictors of Reverse Mismatch in Visually Nonsignificant Lesions (422 lesions)

	Univariate Analysis			Multivariate Analysis		
	OR	95% CI	*P* Value	OR	95% CI	*P* Value
Age, y	0.98	0.96 to 1.00	0.087	0.98	0.95 to 1.01	0.194
Male	2.47	1.30 to 4.68	0.006	2.99	1.44 to 6.23	0.003
ACS nonculprit lesion	2.57	1.48 to 4.45	0.001	2.68	1.40 to 5.12	0.003
LDL‐C, mg/dL	0.99	0.99 to 1.00	0.133	0.99	0.98 to 1.00	0.017
LAD	6.03	3.15 to 11.57	<0.001	6.43	3.07 to 13.47	<0.001
QCA‐RD, mm	0.31	0.20 to 0.46	<0.001	0.34	0.22 to 0.52	<0.001
QCA‐DS, %	1.06	1.03 to 1.08	<0.001	1.06	1.03 to 1.09	<0.001
QCA‐LL, mm	1.04	1.01 to 1.08	0.019	1.03	0.99 to 1.06	0.121
CFR	0.86	0.73 to 1.01	0.071	0.77	0.63 to 0.95	0.017
IMR	0.97	0.95 to 0.99	0.011	0.97	0.94 to 0.99	0.012

ACS indicates acute coronary syndrome; CFR, coronary flow reserve; IMR, the index of microcirculatory resistance; LAD, left anterior descending artery; LDL, low‐density lipoprotein; MI, myocardial infarction; QCA‐DS, diameter stenosis assessed by QCA; QCA‐LL, lesion length assessed by QCA; QCA‐RD, reference diameter assessed by quantitative coronary angiography (QCA).

**Table 6:** Predictors of Mismatch in Visually Significant Lesions (427 Lesions)

	Univariate Analysis			Multivariate Analysis		
	OR	95% CI	*P* Value	OR	95% CI	*P* Value
Diabetes mellitus	0.71	0.45 to 1.10	0.127	0.96	0.58 to 1.60	0.877
Current smoking	1.96	1.22 to 3.15	0.005	1.35	0.75 to 2.43	0.315
RCA	2.90	1.77 to 4.73	<0.001	2.07	1.17 to 3.63	0.012
QCA‐RD, mm	2.37	1.70 to 3.30	<0.001	2.28	1.54 to 3.39	<0.001
QCA‐DS, %	0.93	0.90 to 0.96	<0.001	0.92	0.88 to 0.96	<0.001
QCA‐LL, mm	0.96	0.92 to 0.99	0.005	0.94	0.90 to 0.99	0.009
CFR	1.66	1.40 to 1.97	<0.001	1.62	1.33 to 1.97	<0.001
IMR	1.01	1.00 to 1.02	0.185	1.02	1.00 to 1.04	0.015

CFR indicates coronary flow reserve; IMR, the index of microcirculatory resistance; QCA‐DS, diameter stenosis assessed by QCA; QCA‐LL, lesion length assessed by QCA; QCA‐RD, reference diameter assessed by quantitative coronary angiography (QCA); RCA, right coronary artery.

## Discussion

The main findings of the present study were as follows: (1) QCA‐derived visual and FFR‐defined functional discordance were not rare, with frequencies of mismatch and reverse mismatch being 21.1% and 15.2%, respectively; (2) both mismatch and reverse mismatch were related to the clinical characteristics, lesion‐specific factors, and microvascular resistance; (3) high hyperemic microvascular resistance and high CFR were significant predictors of mismatch; and (4) low hyperemic microvascular resistance and low CFR were significant predictors of reverse mismatch. To the best of our knowledge, this is the first study showing that microvascular function affects both visual‐functional mismatch and reverse mismatch by using IMR as a specific marker of microvascular resistance. Invasive coronary angiography remains a gatekeeper for decision‐making in revascularization because the utilization of FFR is still low, despite the recommendations in the guidelines.^23^ The relationship between angiographic stenosis severity and functional significance assessed by FFR and the mismatch between these 2 measures have been extensively investigated to optimize percutaneous coronary intervention (PCI).^16^, ^24^, ^25^, ^26^ Clinical factors, including age, sex, angiographical lesion‐related factors, and physiological factors, have been reported to contribute to visual‐functional mismatch and reverse mismatch.^15^, ^16^, ^26^, ^27^, ^28^ Our results were in line with these previous studies and further suggested the importance of microvascular function in both mismatch and reverse mismatch. These findings might be related to the fact that FFR as an index is affected by coronary flow volume through the stenosis. Decreased hyperemic microvascular resistance implies that coronary flow might increase for a fixed stenosis and a given driving pressure, and it may dictate a low FFR value despite relatively low resistance to coronary flow induced by the epicardial stenosis and microvascular resistance. In contrast, with increased microvascular resistance, coronary flow through the given fixed stenosis will decrease and FFR will increase without alteration in epicardial resistance to flow resulting from the epicardial stenosis.

### Microvascular Resistance and Functional Stenosis Significance

Although the unequivocal benefit of FFR‐guided revascularization over angiographic guidance has been established,^9^, ^10^, ^11^ it should be noted that FFR is determined under the assumption of minimal and constant microvascular resistance. Our results suggest that microvascular resistance independently and significantly influences visual‐functional mismatch and reverse mismatch in a large study population with intermediate epicardial coronary stenosis. Because FFR relies on a translesional hyperemic pressure gradient in a restricted model of coronary physiology, it is inevitably influenced by pharmacologically induced hyperemic coronary flow or by the individual's responsiveness to vasodilator drugs.^15^ The most important factor related to outcome in patients with coronary heart disease is the presence or absence of inducible myocardial ischemia and its extent. It has been proposed that the presence of microvascular dysfunction is not an obstacle for making decisions based on FFR, provided that minimal and constant microvascular resistance with induced hyperemia is deemed to be nonreversible or consistent before and after revascularization.^28^, ^29^ However, several studies reported the evidence of serial changes in adenosine‐induced microvascular resistance before, after, and during follow‐up post‐PCI.^13^, ^30^, ^31^ van de Hoef et al also reported that identification of epicardial disease severity by FFR is partly obscured by microvascular resistance and that FFR increased with increasing hyperemic microvascular resistance to epicardial disease of equivalent severity.^15^ Our results shed light on the relationship between FFR and microvascular function and indicate that low hyperemic microvascular resistance most likely influences reverse visual‐unctional mismatch for intermediate epicardial stenoses, and that increased microvascular resistance affects visual‐functional mismatch. These findings suggest that the mismatch group might include, at least in part, lesions with low pressure gradients attributed to the decreased coronary flow as a consequence of impaired microvascular resistance.^32^ In other words, the present study suggests that FFR >0.80 and QCA‐DS >50% not only identify vessels without the need for revascularization, but also include vessels with impaired and nonimpaired coronary flow. This potential abnormality in coronary physiology beyond the FFR assumption might provide reasons for why patients with revascularization deferral in randomized trials were not free from long‐term cardiac events, such as in DEFER and FAME follow‐up data, why 12% of patients with FFR >0.80 required revascularization within 2 years of deferral^9^, ^10^, ^11^ and, conversely, why some patients with FFR ≤0.80 but with preserved CFR have a low rate of major adverse cardiac events at follow‐up.^33^

### CFR and Functional Stenosis Significance

In the present study, in addition to IMR, CFR was associated with the FFR value. CFR is the ratio of maximum stress flow to rest flow for the artery of interest; this fundamentally represents coronary flow capacity modified by the integration of epicardial stenosis, diffuse arterial narrowing, and microvascular dysfunction.^32^ Our results demonstrated that CFR positively correlated with the FFR value in the linear regression analysis. Furthermore, a high CFR value was a significant predictor of visual‐functional mismatch in visually significant lesions, and low CFR value was a significant predictor of reverse mismatch in visually nonsignificant lesions. Because CFR is likely to be composed of 3 elements (epicardial stenosis, diffuse narrowing, and microvascular function) and FFR represents the functional significance of epicardial stenosis, our results suggest that epicardial stenosis might influence a significant portion of CFR, rather than diffuse narrowing or microvascular function, in the setting of visual‐functional mismatch evaluation because CFR and FFR showed an independent, parallel correlation.

### Microvascular Resistance, Coronary Flow, and Functional Stenosis Significance

In the present study, a non‐negligible proportion for whom the FFR value was >0.80 showed abnormal coronary microvascular resistance (IMR >28.0; 135 of 472; 28.6%) and disturbed coronary hemodynamics (CFR <2.0; 96 of 472; 20.3%). In those lesions, hyperemic coronary flow might increase if the increased resistance is reduced after revascularization, as suggested by our recent studies.^13^, ^30^ The exact mechanism and pathophysiology of increased or decreased hyperemic microvascular resistance were not known and the change in microvascular resistance and its effect on absolute coronary flow after revascularization remain elusive. Further studies are needed to elucidate the relationship between microvascular resistance and functional stenosis significance by considering the effect of revascularization and the change in microvascular resistance on absolute coronary flow and myocardial ischemia.

### Clinical Implication of Mismatch and Reverse Mismatch

Although frequency and determinants of visual‐functional mismatch have been investigated in the present and previous studies,^15^, ^16^ clinical implication of those lesions has not been elucidated. Specifically, clinical outcomes of the lesions with mismatch or reverse mismatch have not been sufficiently investigated, and, moreover, best therapeutic strategies for those lesions have not been well discussed. If we follow the data from the FAME and FAME2 trials,^10^, ^11^ the mismatch group should be treated by optimal medical therapy alone and the reverse‐mismatch group should be treated by PCI and optimal medical therapy. However, given the substantial population of patients showing FFR within the gray zone ranging from 0.75 to 0.80 in the reverse mismatch group, superiority of PCI for those lesions may deserve further consideration.^34^ Previous studies have suggested the impact of plaque characteristics on FFR values for a given anatomical stenosis in an epicardial coronary artery.^35^ The present study reports the association of CFR and IMR with FFR. Although these factors might be represented by a low FFR value as a single variable, nevertheless, integrated information on patient characteristics, coronary flow, and plaque morphology may provide additive predictive values on FFR, which might enable tailored strategies for lesions, such as gray zone lesions, in which the prognostic values of FFR might be suboptimal.

### Study Limitations

The results of the present study should be interpreted with the consideration of some limitations. First, this was a retrospective study performed at a single center without a dedicated core laboratory for angiographic analysis. Exclusion of patients with significant left main disease, renal impairment, heart failure, or acute coronary syndrome may have resulted in selection bias. This study enrolled patients with stable angina pectoris based on symptoms and noninvasive test results and those who were referred to the catheter laboratory for treatment or diagnosed by diagnostic catheterization at our institution, which might inevitably involve referral bias. There was no clinically validated or normal range of IMR. Therefore, the quartiles of IMR in the study population were used to define high (highest quartile) and low (lowest quartile) IMR. Although this approach provides a reasonable estimation of an abnormal IMR range for patients with coronary heart disease, our results should be tested in an independent study population. Coronary wedge pressure was not measured because this study cohort included lesions that were not treated with PCI, which might have led to overestimation of IMR in tight stenoses with significant collateral flow.^36^

## Conclusions

The present data, which are in line with those of previous studies, indicate that coronary angiography underestimates or overestimates physiological stenosis severity in comparison with FFR in non‐negligible proportion of visually intermediate lesions. In addition, our results indicate that these visual‐functional mismatch and reverse mismatch are related to coronary flow and microvascular function, which may emphasize the importance of coronary flow assessment and coronary pressure indices.

## Disclosures

None.
